# Ulinastatin to prevent acute kidney injury after cardiopulmonary bypass surgery: does serum creatinine tell the whole story?

**DOI:** 10.1186/s13054-016-1356-8

**Published:** 2016-07-01

**Authors:** Patrick M. Honore, Herbert D. Spapen

**Affiliations:** ICU Department, Universitair Ziekenhuis Brussel, Vrije Universiteit Brussel, 101, Laarbeeklaan, Brussels, 1090 Belgium

Ulinastatin is a protease inhibitor derived from human urine with strong anti-inflammatory and anticoagulant activity [1]. Besides its potential to protect tissues against neutrophil-mediated injury, ulinastatin is also anticipated to benefit patients undergoing cardiopulmonary bypass (CPB) surgery by preventing or attenuating post-pump organ injury. Wan et al. recently showed that ulinastatin, given as a 500,000 IU bolus after induction of anesthesia, was associated with a lower incidence of acute kidney injury (AKI) and less need for renal replacement therapy (RRT) after cardiac surgery [2]. This study certainly fills a gap because “renal failure” was barely documented before as a postoperative complication in this patient population [3]. Although data are convincing and shortcomings neatly discussed, we feel that some important aspects remain underexposed. First, the authors do not comment on peri-operative fluid administration. AKI was defined according to the Kidney Disease Improving Global Outcomes criteria using serum creatinine (SCr) but not urine output as a key determinant. SCr, however, has poor specificity and sensitivity for identifying incipient AKI. AKI misclassification may indeed result from flawed estimation of baseline SCr or changes in SCr concentrations due to shifts in fluid balance. For instance, ample fluid loading in the immediate post-operative period after CPB surgery may “dilute” creatinine concentrations and thus mask AKI. Second, mean arterial pressure (MAP) and transfusion amount are independent risk factors for postoperative AKI in cardiac surgery patients [4]. No information is provided with regard to incidence or duration of any episodes of hypotension in either study groups despite MAPs that were near the “critical” 60 mmHg perfusion pressure limit. Patients in the treatment group also tended to receive more red blood cell transfusion. Avoidance of transfusion, especially when hemoglobin levels exceed 8 g/dL, is known to lower the risk for developing AKI in patients undergoing CPB surgery [4]. Third, patients in the treatment group required less RRT but had higher mortality, suggesting that the higher incidence of RRT in controls might reflect severity of illness rather than occurrence of AKI. Finally, a very recently published large controlled trial in patients undergoing elective valve replacement with CPB could not confirm a renal protective effect of ulinastatin [5]. Also, before promoting a more widespread use of ulinastatin, its effects in a predominantly non-Asian population as well as a standard dosing guideline should be better established.

## Abbreviations

AKI, acute kidney injury; CPB, cardiopulmonary bypass; MAP, mean arterial pressure; RRT, renal replacement therapy; SCr, serum creatinine
